# Clinical monitoring and correlates of nephropathy in SIV-infected macaques during high-dose antiretroviral therapy

**DOI:** 10.1186/1742-6405-8-3

**Published:** 2011-01-21

**Authors:** Brigitte E Sanders-Beer, Yvette Y Spano, Dawn Golighty, Abigail Lara, Diane Hebblewaite, Lourdes Nieves-Duran, Lowrey Rhodes, Keith G Mansfield

**Affiliations:** 1 Southern Research Institute, Frederick, MD, USA; 2 Harvard Medical School, New England Primate Research Center, Southborough, MA, USA; 3 BIOQUAL, Inc., 9600 Medical Center Drive, Rockville, MD 20850, USA; 4 Vaccine Research Program, Division of AIDS/NIAID/NIH, 6700B Rockledge Drive, Bethesda, MD 20817, USA

## Abstract

**Background:**

In many preclinical AIDS research studies, antiretroviral therapy (ART) is administered to experimentally simian immunodeficiency (SIV)-infected rhesus macaques for reduction of viral load to undetectable levels. Prolonged treatment of macaques with a high dose of PMPA (9-[2-(r)-(phosphonomethoxy) propyl] adenine or tenofovir; 30 mg/kg of body weight subcutaneously once daily) can result in proximal renal tubular dysfunction, a Fanconi-like syndrome characterized by glucosuria, aminoaciduria, hypophosphatemia, and bone pathology. In contrast, chronic administration of a low dose of PMPA (10 mg/kg subcutaneously once daily) starting at birth does not seem to be associated with any adverse health effects within 3 years of treatment. In contrast to PMPA, limited information on systemic toxicity in rhesus monkeys is available for FTC (5-fluoro-1-(2R,5S)-[2-(hydroxymethyl)-1,3-oxathiolan-5-yl]cytosine; emtricitabine) and stavudine (d4T).

**Results:**

In this study, the clinical and biochemical correlates of tubular nephrosis in SIV-infected rhesus macaques associated with systemic administration of high-dose ART consisting of the three nucleoside analog inhibitors PMPA, FTC, and d4T were investigated. It was found that acute renal failure was uncommon (7.1% of treated animals) and that morphologic evidence of nephropathy, which persisted for more than 300 days following discontinuation of the drug cocktail, was more frequent (52.4% of treated animals). While parameters from single time points lacked predictive value, biochemical alterations in Blood Urea Nitrogen (BUN) and phosphorus were frequently identified longitudinally in the blood of ART-treated animals that developed evidence of nephropathy, and these longitudinal changes correlated with disease severity.

**Conclusions:**

Recommendations are proposed to limit the impact of drug-induced renal disease in future SIV macaque studies.

## Background

The nucleotide reverse transcriptase inhibitor (NRTI) PMPA or tenofovir has become one of the most commonly used antiretroviral drugs due to its favorable efficacy and safety profile, based on data collected over more than 9 years for HIV-infected adults. The acyclic nucleoside phosphonate PMPA is renally excreted by a combination of glomerular filtration and active tubular secretion [1]. The effective uptake of acyclic nucleoside phosphonates by organic anion transporters in proximal tubules leads to accumulation in tubular cells and dose-limiting toxicity in animals [2]. Renal toxicity is usually manifested as renal insufficiency and proximal renal tubular dysfunction (PRTD). FTC or emtricitabine is the (-) enantiomer of a thio analog of cytidine, which differs from other cytidine analogs in that it has a fluorine in the 5-position. It is another nucleoside analog HIV-1 reverse transcriptase inhibitor and also mainly eliminated by the kidney. ZERIT^® ^is the brand name for d4T or stavudine, a synthetic thymidine nucleoside analogue. D4T is phosphorylated by cellular kinases to the active metabolite d4T triphosphate, which inhibits the activity of HIV-1 reverse transcriptase (RT) by competing with the natural substrate thymidine triphosphate and by causing DNA chain termination following its incorporation into viral DNA. D4T triphosphate inhibits cellular DNA polymerases β and γ and markedly reduces the synthesis of mitochondrial DNA. Urinary excretion is the major route of d4T elimination.

Although systemic ART is clearly of benefit, a variety of antiretroviral drugs including protease inhibitors and NRTIs, have been linked to nephrotoxicity [3]. SIV-infected macaques also develop renal disease that mimics the scope and etiology of that observed in HIV-infected people. Rhesus macaques develop opportunistic infections such as SV40, cytomegalovirus and adenovirus infection that may produce renal pathology and resemble the disease processes recognized in HIV-infected patients. Furthermore, a segmental glomerulosclerosis has been described in SIV-infected animals that have progressed to AIDS, which is similar morphologically to the HIV-nephropathy observed in human patients [4-6]. Finally macaques may also develop renal dysfunction subsequent to antiretroviral therapy with PMPA [7-11]. PMPA is the biologically active metabolite of the prescription drug Viread^®^. It is commonly used in SIV pathogenesis studies because it can be administered by the parenteral route and is highly effective at reducing viral loads. Previous work of others has revealed that long-term administration of PMPA at 30 mg/kg resulted in a Fanconi-like syndrome with glucosuria, aminoaciduria, hypophosphatemia, growth restriction and bone pathology [2]. In this report, the serum biochemical correlates of renal morphologic alterations in SIV-infected macaques that received PMPA, d4T and FTC combination therapy are described and guidelines to prevent and identify serious renal sequellae in future experiments are proposed.

## Results

### Effectiveness of ART in reducing SIV RNA load

As described in zur Megede et al. [12], ART (PMPA, FTC, and d4T) was administered during the chronic phase of SIV infection (13 weeks post infection (wpi)) and was continued until 41 wpi. Viral load was very efficiently controlled by ART, dropping below the assay detection limit (<200 RNA copies/ml) in most of the animals by 20 wpi, and only rebounded upon discontinuation of ART.

### Acute renal failure may be associated with NRTI-based ART

Thirty-three rhesus macaques (*Macaca mulatta*) were initially enrolled in the study [12]. Treatment groups and disease outcomes are provided in Table 1. Of these animals, 30 were inoculated with SIVmac239 and 28 received antiretroviral treatments consisting of PMPA, FTC, and d4T, which was highly effective in controlling viral replication in the majority of cases. Two animals were rapid progressors and had to be euthanized due to the development of AIDS-like symptoms at 8 and 13 wpi, respectively (3445, 3529). Another two animals were euthanized 4 and 5 weeks after start of ART treatment, respectively, due to opportunistic infections (3406, 3448), and one animal died from cardiac arrest during anesthesia (3528). At week 22, another six animals were excluded from the study due to incomplete virus control under ART (3443, 3517, 3519, 3520, 3521, 3523). The remaining 19 animals, including nine MamuA01+ animals, were randomly distributed into three groups according to their MamuA01 status and viral load. Seven animals received ART only (3447, 3451, 3452, 3453, 3511, 3515, 3684), in six animals immunization with SIV DNA by IM electroporation was conducted four times in four-week intervals (3444, 3522, 3524, 3526, 3527, 3530), and six animals additionally received 50,000 IU/kg IL-2 administered twice daily by the subcutaneous route from day 2-16 following immunization (3449, 3512, 3514, 3516, 3518, 3525). Of the 28 animals that received PMPA, two (3406 and 3448) had biochemical evidence of acute renal failure that developed 26 and 31 days, respectively, following initiation of ART. Abnormalities included marked and abrupt increases in serum BUN, creatinine and calcium (Table 1). Four animals were euthanized because of continuing high virus replication or onset of SIV-related disease and, although tissues were not evaluated, they did not have biochemical evidence of renal failure. One animal died peracutely, and autolysis prevented interpretation of tissue samples. The remaining 21 animals were followed by sequential evaluation of serum chemistries, and renal tissue was evaluated morphologically upon euthanasia. None of these animals developed biochemical evidence of acute or chronic renal failure. Histologically, acute renal failure was documented in 7.1% (2/28) of the animals receiving ART, for which suitable samples were available for analysis. Both cases occurred during the administration of high-dose PMPA (30 mg/kg) in combination with FTC and d4T and resulted in rapidly progressive and nonreversible acute renal failure.

**Table 1:** Summary of animal groups and disease outcomes

Animal ID	Age (yrs)*	Weight (kg)*	MHC type	Reason for death	Survival (months)	SIV	ART Treatment	ART nephropathy	Renal failure
3406	14.0	13.5	A02	nephropathy	4	yes	ART	likely	BUN 181, Ca/P 15.6/9.6, creatinine 23.3
3442	5.0	8.7	B01	open	11	no	-	n/a	n/a
3443	5.9	8.3	-	autolysis	14	yes	ART	unknown	unknown
3444	5.5	10.9	A01, B01	study ended/euthanized	18	yes	ART+DNA	yes	no biochemical evidence
3445	5.2	7.5	A08	open	2	yes	-	n/a	n/a
3446	7.3	9.3	A08	n/a	n/a	no	-	n/a	n/a
3447	6.3	11.5	A01, A08	study ended/euthanized	18	yes	ART	yes	no biochemical evidence
3448	5.4	5.7	-	nephropathy	4	yes	ART	likely	BUN 113, Ca/P12.9/4.8, creatinine 6.4
3449	6.6	13.8	A08	study ended/euthanized	18	yes	ART+DNA+IL-2	yes	no biochemical evidence
3451	8.6	10.0	-	study ended/euthanized	18	yes	ART	yes	no biochemical evidence
3452	7.5	9.3	A08	study ended/euthanized	18	yes	ART	yes	no biochemical evidence
3453	6.8	8.8	-	study ended/euthanized	18	yes	ART	no	no biochemical evidence
3511	5.4	9.2	A01	study ended/euthanized	20	yes	ART	no	no biochemical evidence
3512	5.3	6.7	A02	granulomatous hepatitis	17	yes	ART+DNA+IL-2	no	no biochemical evidence
3514	5.3	9.0	A01, A08, B01	study ended/euthanized	19	yes	ART+DNA+IL-2	yes	no biochemical evidence
3515	5.4	10.2	A01	study ended/euthanized	19	yes	ART	yes	no biochemical evidence
3516	5.4	10.1	A01, A08	study ended/euthanized	19	yes	ART+DNA+IL-2	yes	no biochemical evidence
3517	4.7	5.2	-	poor viral control	10	yes	ART	unknown	no biochemical evidence
3518	5.4	8.9	-	study ended/euthanized	19	yes	ART+DNA+IL-2	yes	no biochemical evidence
3519	5.4	7.4	A02, A08, B01	study ended/euthanized	18	yes	ART	no	no biochemical evidence
3520	4.6	4.4	A08, B01	poor viral control	10	yes	ART	unknown	no biochemical evidence
3521	4.7	5.9	A01, B01	poor viral control	10	yes	ART	unknown	no biochemical evidence
3522	5.5	7.2	A01	study ended/euthanized	20	yes	ART+DNA	no	no biochemical evidence
3523	5.2	6.9	A08	SIVE/Pneumonia	17	yes	ART	no	no biochemical evidence
3524	5.5	9.2	A01	study ended/euthanized	19	yes	ART+DNA	yes	no biochemical evidence
3525	5.4	8.3	A01, B01	study ended/euthanized	19	yes	ART+DNA+IL-2	no	no biochemical evidence
3526	5.7	6.5	A08, B01	study ended/euthanized	20	yes	ART+DNA	no	no biochemical evidence
3527	5.7	10.7	A08	study ended/euthanized	20	yes	ART+DNA	no	no biochemical evidence
3528	4.6	5.5	A01, B01	diseased	5	yes	ART	unknown	no biochemical evidence
3529	4.4	5.1	A08	open	3	yes	-	n/a	n/a
3530	5.3	10.7	A02, B01	study ended/euthanized	19	yes	ART+DNA	yes	no biochemical evidence
3680	8.8	14.4	B01	n/a	n/a	no	-	n/a	n/a
3684	6.2	10.9	B01	pneumonia	14	yes	ART	yes	no biochemical evidence

*at study end

### Morphologic evidence of ART nephropathy is common and persistent

**Figure 1:**
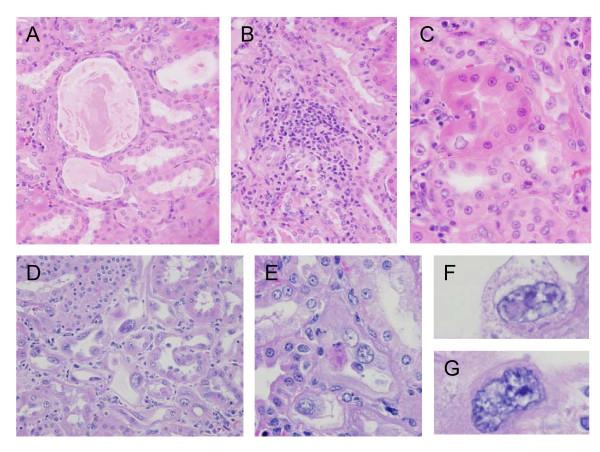
**Morphologic features of ART nephropathy**. Ectasia of renal tubules containing eosinophilic proteinaceous material (tubular proteinosis) (A) and focal lymphocytic infiltrate (B). Mild cytomegaly and anisonucleosis of proximal convoluted tubule (PCT) (C). Marked nuclear dysplasia with mild cytomegaly and tubular ectasia (D). Focal necrosis of PCT epithelial cell (E). Nuclear dysplasia with cytoplasmic inclusion and nuclear vesiculation (F) and early lobulation (G).

Twenty-one cases were submitted for necropsy evaluation and were suitable for microscopic evaluation of renal morphology. The range of survival following completion of ART for these animals was from 122 to 300 days. Three NHP were euthanized for health-related reasons prior to study end, and 18 animals were euthanized at the end of the study. 52.4% (11/21) of these animals developed morphologic evidence of ART nephropathy. A variety of changes were observed in effected animals (Figure 1): 1) nuclear dysplasia of proximal convoluted tubular (PCT) epithelium (anisonucleosis, meganucleosis, nuclear lobulation, heterochromaisa and/or cytoplasmic invaginations) (100.0%); 2) interstitial fibrosis (91.6%); 4) PCT basophilia (75.0%); 5) tubular proteinosis (75.0%); 6) ongoing tubular necrosis (75.0%); 7) cytomegaly of PCT (66.7%); 8) interstitial nephritis (50.0%); and 9) cellular casts (8.3%).

Since all NHP were male, there was no correlation with sex (Table 1). The NHP that were diagnosed with nephropathy (n = 12; mean age 6.1 years) were slightly older than the animals without nephropathy (n = 9; mean age 5.6 years), but the difference was not statistically significant (p = 0.22; two tailed t-test). In contrast, the NHP that were diagnosed with nephropathy (n = 12; mean body weight 10.4 kg) were slightly heavier than the animals without nephropathy (n = 9; mean body weight 8.0 kg), and the difference was statistically significant (p = 0.0008; two tailed t-test). No obvious correlation between development of nephropathy and the MHC types MamuA01, A02, A08, and B01 could be detected (Table 1). No differences were observed in those animals receiving IL-2 as a component of their vaccine regimen (Table 1). These findings are consistent with alterations previously described in both humans and macaques with ART nephropathy. Nuclear changes observed in the PCTs were unique and have not been observed outside the context of ART nephropathy in SIV-infected macaques. While morphologic evidence of ART nephropathy was frequent, clinical disease evidenced by overt azotemia or renal failure was not observed in animals following discontinuation of drug.

### Biochemical differences in serum are observed frequently at different stages of treatment and disease

**Figure 2:**
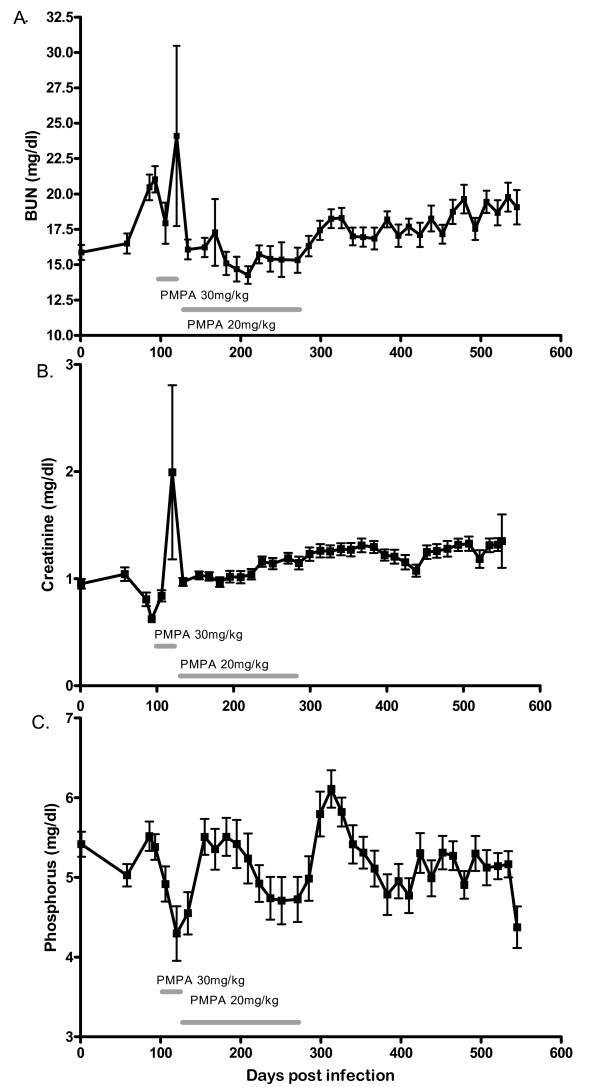
**Longitudinal changes in serum BUN, creatinine and phosphorus following SIV infection and ART treatment**. Longitudinal changes in Blood Urea Nitrogen, creatinine, and phosphorus in the serum of ART-treated, SIV-infected rhesus macaques. Shown are mean and standard deviation for a period of 79 weeks after SIV infection. Durations of PMPA treatment with the 30 mg/kg and 20 mg/kg dose are indicated.

**Figure 3:**
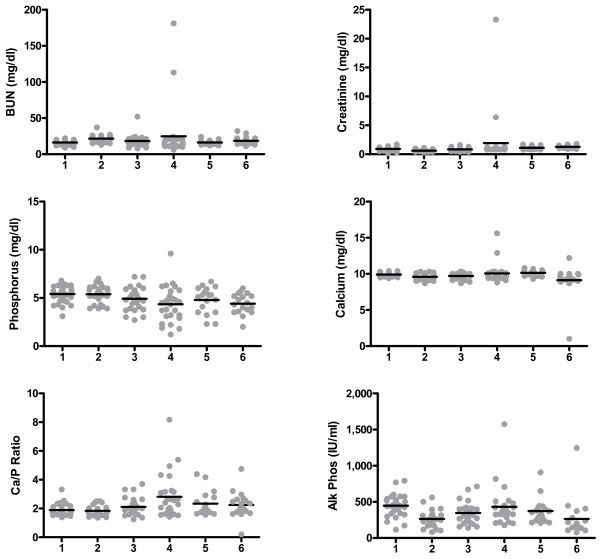
**Alterations in serum chemistry values at defined time points during the course of treatment and disease**. Alterations in blood urea nitrogen, creatinine, phosphorus, calcium, the Ca/P ratio and alkaline phosphatase were plotted at critical time points (1 = baseline, 2 = initiation of ART, 3 = 2 weeks ART, 4 = 4 weeks ART, 5 = ART end, 6 = study end). Grey dots represent individual animals and black lines represent the mean.

Longitudinal changes in serum chemistry were examined at different stages of disease and treatment and revealed an initial increase in BUN and creatinine and decrease in phosphorus, which coincided with ART initiation (Figure 2). These values tended to normalize following PMPA dose reduction from 30 to 20 mg/kg. Following discontinuation of ART, phosphorus increased further, and there were upward trends in both creatinine and BUN. One-way analysis of variance (Anova) was used to compare values at base line, initiation of ART, 2 weeks of ART, 4 weeks of ART, termination of ART, and end of study (Table 2 and Figure 3). Differences were observed for calcium (p < 0.001), phosphorus (p = 0.0042), alkaline phosphatase (p < 0.0001), Ca/P (p < 0.001), creatinine/phosphorus (p < 0.0001), and BUN/phosphorus (p < 0.001). A post (ANOVA) test pairwise comparison was performed to examine differences at select time points with the result that statistically significant increases in the calcium/phosphorus ratio (p < 0.001), the creatinine/phosphorus ratio (p < 0.001), and alkaline phosphatase (p < 0.05), and a statistically significant decrease in phosphorus (p < 0.05) were present after 4 weeks of ART compared to values obtained at the initiation of ART (Table 2). Further differences were not observed at the termination of ART suggesting that only the higher dose of PMPA was associated with adverse outcomes or that compensatory mechanisms had come into play for reducing serum chemistry changes.

**Table 2:** Comparison of Ca, P, Ca/P, BUN/P, Creatinine/P and alkaline phosphatase levels at select time points during treatment and disease

		Initiation ART vs 2 wks ART	Initiation ART vs 4 wks ART	Initiation ART vs ART end	Initiation ART vs study end
Ca	Mean Diff.	0.1222	0.4667	0.5548	-0.4759
	P value				
					
P	Mean Diff.	-0.4593	-1.037	-0.5944	-0.9722
	P value		**P < 0.05**		
					
Ca/P	Mean Diff.	0.2767	0.9773	0.5025	0.4104
	P value		**P < 0.001**		
					
BUN/P	Mean Diff.	-0.237	1.844	-0.3889	0.4556
	P value				
					
Crea/P	Mean Diff.	0.07223	0.3088	0.1579	0.1963
	P value		**P < 0.001**		
					
Alk Phos	Mean Diff.	-82.33	167.4	107.9	0.6296
	P value		**P < 0.05**		

### Individual serum chemistry values lack predictive value of chronic disease

**Figure 4:**
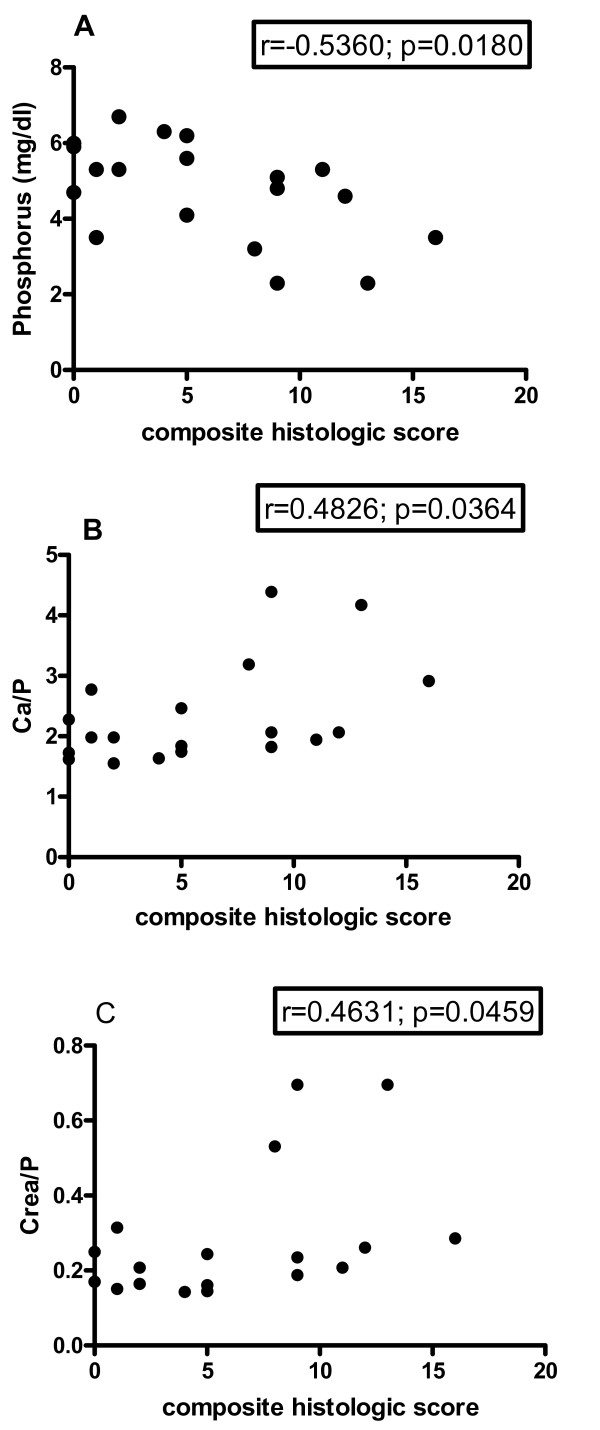
**Correlation of serum phosphorus level, Ca/P ratio and Crea/P ratio at the end of ART with severity of nephropathy**. Correlation between composite histology score (see criteria in Table 3) and phosphorus (A)/Ca-P ratio (B)/Crea-P ratio (C). A composite ART nephropathy score was generated through the addition of individual tubular pathology values (see Table 3). The p values for the individual correlations are shown next to the slope "r".

**Figure 5:**
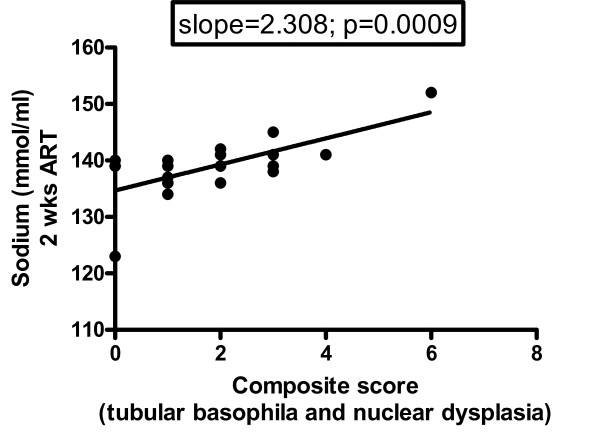
**Relationship between serum sodium levels at 2 wks of ART and ongoing pathology within the proximal convoluted tubules at death**.

To determine whether serum chemistry values were predictive of the development of chronic ART nephropathy, values were compared to individual and composite histologic scores. No statistically significant correlations were observed at base line, initiation of ART, 2 weeks ART, or 4 weeks ART for BUN, creatinine, phosphorus, calcium, albumin, globulin, glucose or alkaline phosphatase. At discontinuation of ART a negative correlation was observed between histologic score and phosphorus (r = -0.5360; 95% CI -0.7964 to -0.1079; p = 0.018) and positive correlations were observed with Ca/P (r = 0.4826; 95% CI 0.324 to 0.7684; p = 0.0364) and Crea/P (r = 0.4631; 95% CI 0.1109 to 0.7579; p = 0.0459) (Figure 4). At study termination, a positive correlation was observed between creatinine and composite histologic score (r = 0.4947; 95% CI 0.0372 to 0.7817; p = 0.037). Examination of individual histologic parameters revealed an unexpected relationship between serum sodium levels at 2 wks of ART and ongoing pathology in the proximal convoluted tubules at death (slope = 2.308; p = 0.0009) (Figure 5). This suggests that factors influencing hydration during treatment regimen may impact disease course.

While differences were observed within groups as a whole, values obtained from individual animals during ART were of limited use in predicting those animals that would develop morphologic alterations of disease. Since only two animals developed acute renal failure, it is difficult to determine the predictive value of serum chemistry parameters for severe disease. Alterations in BUN and creatinine were only observed within 4 days of death suggesting that they lack sensitivity and do not represent useful predictive markers. A mild hypophosphatemia was observed in one animal approximately two weeks prior to death, but was not observed in the second animal.

### Longitudinal changes in serum chemistry values correlate with chronic disease

**Figure 6:**
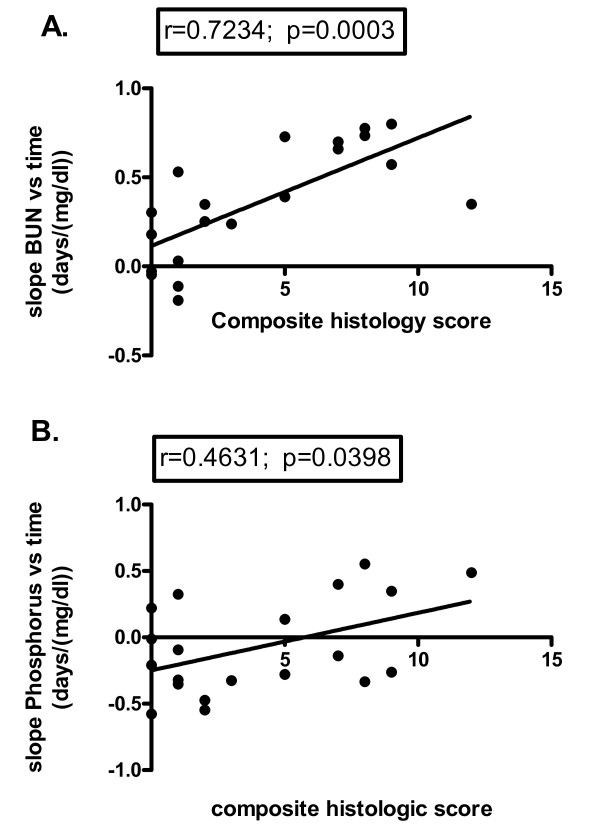
**Correlation of BUN and phosphorus slope with composite ART nephropathy score**. Correlation of BUN (r = 0.7234; p = 0.0003) and phosphorus (r = 0.4631; p = 0.0398) slope with composite ART nephropathy score reveals statistical significance. Black dots represent individual animals.

To determine whether longitudinal alterations in biochemical values were associated with morphologic evidence of ART nephropathy, linear regression was performed for serum chemistry values from individual animals, and the slope of change was compared to composite histologic scores. Positive correlations were observed between composite histologic scores and changes in BUN (r = 0.7234; 95% CI 0.4131 to 0.8832; p = 0.0003) and phosphorus (r = 0.4631; 95% CI 0.02575 to 0.7516; p = 0.0398) (Figure 6). No statistically significant correlation was observed between changes in serum creatinine and composite histologic score. In all, 9 of 21 animals had statistically significant positive slopes for BUN over time. For animals with no evidence of ART nephropathy 11.1% (1/9) had a positive slope compared to 66.7% (8/12) with evidence of ART nephropathy (p = 0.0244; Fischer exact test). These findings indicate that morphologic alterations resulted in biochemical changes consistent with progressive renal disease and suggest that over time some animals may have progressed to renal failure.

## Discussion and Conclusion

The results presented indicate that ART nephropathy was common and persistent in SIV-infected rhesus macaques receiving combination PMPA-containing ART. Animals developed morphologic and biochemical evidence of disease despite normal renal function at the onset of the study. This is in contrast to human patients, in whom disease is said to be infrequent and associated with pre-existing renal pathology. These differences may be related to differences in species, drug or drug dose, and route. The dose of the prodrug tenofovir disoproxyl fumarate (TDF; Viread^®^) in human patients is substantially lower on a per kilogram basis than the equivalent PMPA dose used in rhesus macaques, which in combination with species differences in drug pharmacokinetics results in lower plasma drug levels in humans [13,14]. In addition, while Viread^® ^is given orally in human patients, PMPA is administered as a single subcutaneous injection once daily in macaques, which may further increase peak plasma drug values. In addition, the FTC dose that was used in these studies (50 mg/kg) is also higher than the equivalent regimen in humans. Others demonstrated that a dose of 20 mg/kg of FTC is equivalent to the human dose [15]. This higher dose of FTC may also have contributed to toxicity. However, for d4T, the dose administered for NHP in this study (2.4 mg/kg/day) was slightly lower than the dose recommended for humans, based on a body surface conversion model [16]. Although morphologic evidence of ART nephropathy was frequent, associated acute renal failure was less common (7.1%) and overt chronic renal failure demonstrated by azotemia following completion of the ART regimen was not observed over the time period monitored. Changes in BUN over time suggest that if a longer follow-up period is allowed, a subset of animals might develop chronic renal failure. In the present study, the occurrence of ART nephropathy after termination of treatment did not appear to effect clinical outcome or survival. Nonetheless, subclinical renal dysfunction could impact experimental outcome through alteration of the pharmacokinetics of co-administered drugs, changes in calcium/phosphorus balance or initiation of other sequellae of chronic renal disease. It was also found that animals may have substantial morphologic alterations at the light microscopic level and normal BUN and creatinine levels in serum, reinforcing the relative insensitivity of these tests in diagnosing renal pathology. For these reasons, investigators should be made aware of ART nephropathy, and a better understanding of risk factors associated with its development should be sought.

The toxic effects of PMPA on bone and kidney in SIV-infected macaques have previously been described in detail [2,17]. These findings included growth restriction and biochemical and morphologic features of renal tubular dysfunction, which were frequently observed in animals receiving PMPA at 30 mg/kg for periods exceeding 8 months [2]. The results presented here differ in that short term (30 days) administration of PMPA at this dose was associated with acute renal failure in a small subset of animals and was more frequently associated with morphologic and biochemical evidence of renal dysfunction for up to 300 days following cessation of treatment. The reason for this difference is unknown, and since our cohort received combination therapy, it is possible that co-administration of the other NRTIs, d4T and FTC, may have potentiated the nephrotoxic effects of PMPA [18-21]. This cohort was also substantially older than the neonatal and juvenile animals previously examined. Van Rompay reported that PMPA renal clearance was lower in adult as compared to juvenile animals, and thus age differences may play a role in determining disease susceptibility and course [2]. Lower PMPA clearance may allow for higher plasma concentrations to be achieved and promote drug accumulation within the PCT epithelium, thereby exacerbating renal toxicity. Once renal damage occurs, PMPA clearance may decrease leading to a further reduction in tubular function.

It was found that individual serum chemistry values were of limited use in predicting acute renal or persistent renal pathology. The identified changes were consistent with the proposed pathogenesis of ART nephropathy and changes previously observed in humans and macaques.

Several recommendations are proposed to reduce the impact of ART nephropathy on future studies:

### 1) Consider reduction of the 30 mg/kg PMPA dose or shortening of therapy duration

Cases of acute renal failure were observed during or shortly after completion of the 30 mg/kg dose regimen. Furthermore, biochemical abnormalities observed after the 4 weeks of ART had largely resolved at termination of ART. While this may have been due to compensatory changes, more likely it was the result of the dose reduction. If it is felt that an initial 30 mg/kg dose is needed to achieve adequate virologic control, the duration of the higher dose could be reduced to 2 weeks. This would likely decrease the incidence of acute renal failure and ART nephropathy and may be particularly important in older animals or animals administered other potentially nephrotoxic drugs.

### 2) Defined criteria should be established for discontinuation of ART

Acute renal failure appeared to develop rapidly, and biochemical changes in the serum did not appear until significant renal dysfunction was apparent. Unfortunately, serum chemistry values were of limited value in predicting acute renal failure. Defined biochemical criteria to remove animals from NRTI-based ART should include BUN >30 mg/dl, creatinine >2.0 mg/dl and phosphorus <3.0 mg/dl. Because of the rapidly progressive nature of renal dysfunction in this condition, such criteria may still be of limited use. If acute nephropathy is recognized, concurrent administration of intravenous fluids and judicious use of diuretics should be considered.

### 3) Management of subclinical dehydration

The positive correlation between serum sodium at 2 weeks on ART and the severity of nephropathy at the termination of the study was striking. While individual sodium levels lack predictive value, this finding suggests that mild dehydration during the early phase of treatment may predispose to more severe renal changes. Options to improve hydration during this critical period, such as supplementing animals' water consumption with juice or fruit, should be considered and overt clinical dehydration should be treated aggressively.

### 4) Consider increasing the frequency of serum chemistry evaluation during high dose treatment

Increasing the frequency of serum chemistry evaluation during high dose therapy may increase the ability to detect changes associated with acute renal failure allowing time to discontinue drug and intervene. Use of weekly samples during this time should be considered. Similarly, if there is a means to measure water consumption or urine production during the first four weeks of treatment, this may represent a sensitive measure of impending renal failure.

### 5) Other objective measures that may have predictive value of acute renal failure should also be sought

Because of the limited value of serum chemistries for detection of nephropathy, other measures of renal function should be considered. The collection and analysis of urine may be of some benefit, but defined criteria for drug withdrawal are lacking. Urinary glucose, protein, and specific gravity can be easily and rapidly measured on samples and will likely reveal abnormalities during PMPA treatment. Other measures that might prove useful include urinary β2-microglobulin and urinary protein/creatinine ratio. Point-of-care diagnostic devices are available to facilitate measurement of the latter and might be considered. As with serum chemistry evaluations, increased sample frequency during high dose treatment may be beneficial. While potentially useful, further work will be required to develop objective criteria for drug withdrawal.

## Methods

### Nonhuman Primate Studies

The nonhuman primate study was conducted at Southern Research Institute in Frederick, MD and was approved by the Institutional Animal Care and Use Committee. Briefly, male Indian-origin rhesus macaques were inoculated intravenously with 1,000 TCID_50 _SIVmac239 and followed prospectively with sequential blood draws used for determination of viral load, CD4 T cell numbers, complete blood counts, and serum chemistries. Animals were started on ART 3 months after SIV infection and treated for 6 months. ART consisted of PMPA (20-30 mg/kg/SC SID), d4T (stavudine; 1.2 mg/kg BID PO) and FTC (emtricitabine; 50 mg/kg SC SID). PMPA was given at 30 mg/kg for the first month and then reduced to 20 mg/kg thereafter. During ART, animals received a therapeutic SIV DNA vaccine four times in four-week intervals. A subset of animals also received Proleukin^® ^from day 2-16 after vaccination, and the animals were followed for 75 weeks when the study was terminated [12]. Two animals developed biochemical evidence of acute renal failure during the treatment period, and others subsequently developed morphologic evidence of nephropathy. The purpose of this analysis was to summarize histological findings within renal tissue and examine serum biochemistry correlates of these changes to identify predictors of disease occurrence useful in the management of future studies.

### Histopathology

Paraffin-embedded, formalin-fixed renal tissue from 21 animals was available for histopathology. Sections were cut and routinely stained with hematoxylin and eosin. Objective criteria were developed to provide a measure of renal tubular pathology (Table 3) and were applied to the evaluation of tissues in a blinded fashion. A composite ART nephropathy score was generated through the addition of individual tubular pathology values. Excellent agreement was found between this composite score and a subjective nephropathy score (0, normal; 1 mild; 2, moderate; and 3, severe) (r = 0.9454; p < 0.0001) generated in an independent and blinded fashion.

**Table 3:** Renal pathology grading criteria

Grade	0	1	2	3	4
Tubular protein	normal	present in 1 tubule/lpf	present in 2-3 tubules/lpf	present in 2-3 tubules/lpf; with tubular ectasia	present in >3-4 tubules/lpf

Tubular basophilia	normal	mild	moderate	severe	

Tubular casts	normal	mild	moderate	severe	

Tubular necrosis	normal	necrotic cells present; 1/hpf; scattered tubular atrophy	necrotic cells present; 1-4/hpf; moderate tubular atrophy	necrotic cells present; >4/hpf; extensive tubular atrophy	

Interstitial fibrosis	normal	equivoval	mild fibrosis; <1%	moderate 5-15%	>15%

Cytoplasmic droplets	normal	rarely present	present in 10-20 cells/hpf	present in >20 cells/hpf	

Nuclear dysplasia	normal	anisonucleosis rarely present	anisonucleosis present; lobulated nuclei <5/hpf	anisonucleosis present; lobulated nuclei 5-10/hpf	anisonucleosis present; lobulated nuclei >10/hpf

Interstitial nephritis	normal	<1% of lpf; equivocal necrosis	1-5% of with necrosis	>5% of lpf with necrosis	>20% of lpf with necrosis

Nuclear cytoplasmic invaginations	none	Present			

Cytomegaly	normal	anisocytosis rarely present	present in 5% of PCT cells	present in >5% PCT cells	

### Serum chemistry

Serum chemistry values for BUN, creatinine, phosphorus, calcium, albumin, globulin, sodium, chloride, and alkaline phosphatase were measured using a VetScan Chemistry Analyzer (Abaxis, Inc.) every 2 to 4 weeks. At defined time points (base line (t = 0), initiation of ART (t = 92 days), 2 weeks of ART (105 days), 4 weeks of ART (119 days), termination of ART (284 days), and study end (day of death) values were compared to composite and individual pathology scores using statistical software (GraphPad Prism 4). In addition, linear regression analysis was performed for data sets from individual animals to determine, which animals had statistically significant changes in phosphorus, BUN, and creatinine over time. The slopes of these changes were then compared to composite ART nephropathy scores to determine whether morphologic changes correlated with biochemical changes.

## Competing interests

The authors declare that they have no competing interests.

## Authors' contributions

BES-B and YES designed and supervised the study. DG, AL, DH, and LN-D were carrying out the technical aspects of the study, such as CBC and serum chemistries and RNA viral loads. LR performed the veterinary supervision, clinical observations and therapeutic interventions on the animals. KM conducted the histopathology evaluations and the correlations with serum chemistries. All authors read and approved the final manuscript.
